# Remove Persistent Staining with a Callus Shaver

**DOI:** 10.1097/GOX.0000000000002140

**Published:** 2019-03-25

**Authors:** Apinut Wongkietkachorn, Palakorn Surakunprapha, Vor Luvira, Nuttapone Wongkietkachorn, Supawich Wongkietkachorn

**Affiliations:** From the * Division of Plastic and Reconstructive Surgery, Department of Surgery, Faculty of Medicine, Mae Fah Luang University, Chiang Rai, Thailand; † Division of Plastic and Reconstructive Surgery, Department of Surgery, Faculty of Medicine, Khon Kaen University, Khon Kaen, Thailand; ‡ Department of Surgery, Faculty of Medicine, Khon Kaen University, Khon Kaen, Thailand; § Division of Plastic and Reconstructive Surgery, Department of Surgery, Q Clinic, Bangkok, Thailand; ¶ Department of Surgery, Faculty of Medicine, Thammasat University, Pathum Thani, Thailand.

## Abstract

Supplemental Digital Content is available in the text.

## Sir,

Removing persistent staining from the periwound area can be difficult in patients with poor hygiene. Stain removal is important because these stains can become colonized with bacteria, which can then migrate to the wound.^1,2^ Normally, cleaning of these stains begins with chemical removal using antiseptic soaps and solutions.^3,4^ If chemical removal is not successful, the stain is removed mechanically. The instruments that are usually used in mechanical removal are forceps and a scalpel.^3^ The forceps are used to peel the epidermis, and the scalpel is used to cut the dead skin. However, this process can sometimes be traumatic, as it is difficult to control the peeling depth and the scalpel is sharp and can easily damage the dermis.

Callus shavers, which are used by nonsurgeons to shave the soles of the feet at home, are safe and do not cause trauma (**see figure, Supplemental Digital Content 1**, which displays the callus shaver instrument, http://links.lww.com/PRSGO/B1). We found that a callus shaver could also be used to remove persistent stains through the removal of the keratin layer from the skin. Moreover, this tool is cheap and available in many stores.

**Fig. 1.**
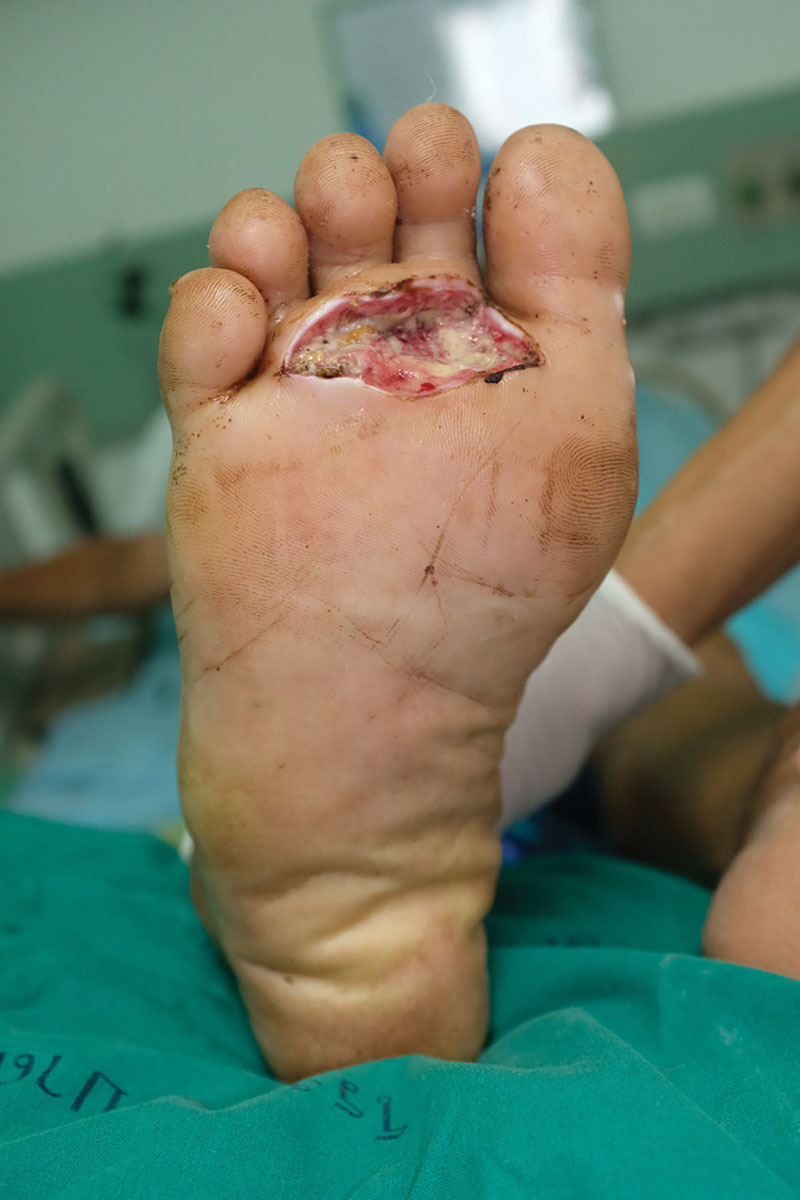
An infected diabetic wound on the right foot with persistent stains at the periwound area.

This procedure was used on a 72-year-old man who was admitted with an infected diabetic foot. His right foot was extremely unhygienic (Fig. 1). Although various chemical solutions (ie, Hibiscrub, Betadine surgical scrub, chlorhexidine, povidone-iodine, and acetone) were used in an attempt to remove the stains in the periwound area, the stains persisted (see **figure, Supplemental Digital Content 2**, which displays the wound after various chemical solutions were used in an attempt to clean the periwound area, but the stains persisted, http://links.lww.com/PRSGO/B2).

**Fig. 2.**
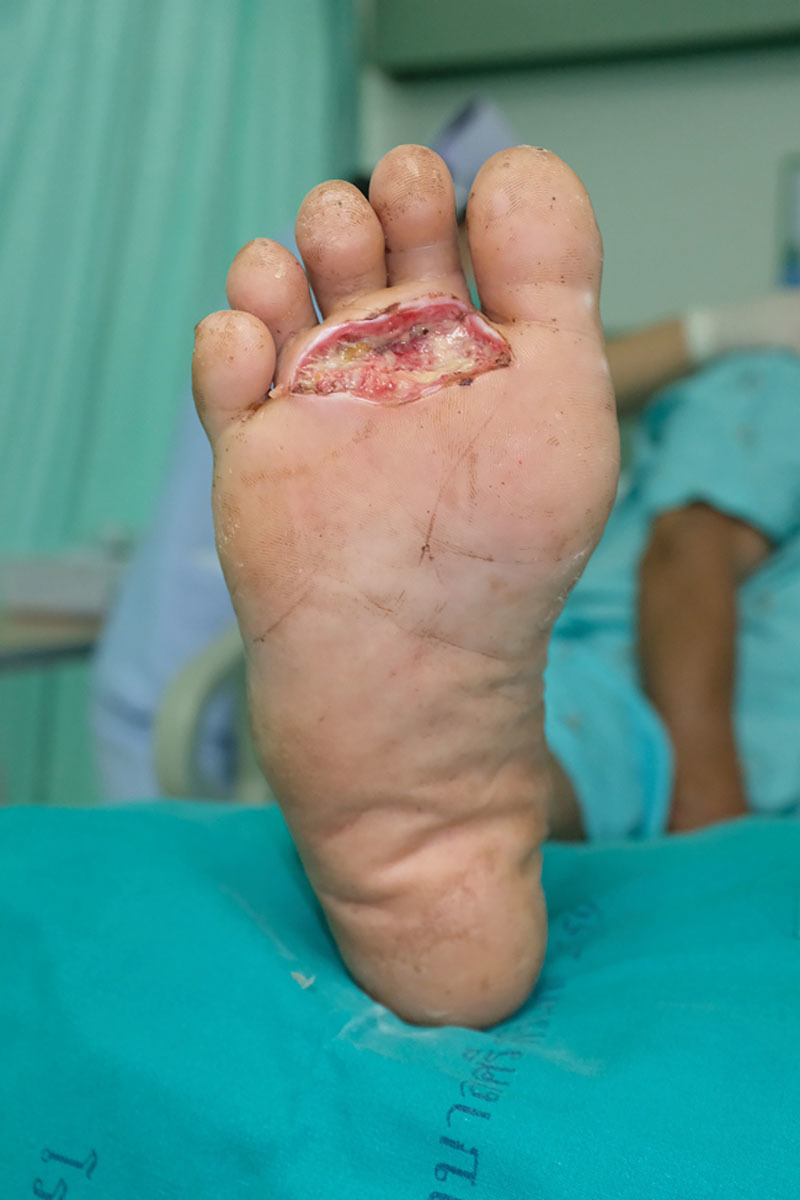
After removal of persistent stains with a callus shaver.

However, a callus shaver was then used and was able to easily remove the remaining stains. The process is shown in Supplemental Digital Content 3, and the results are shown in Figure 2 (**see video, Supplemental Digital Content 3**, which demonstrates how to remove persistent staining with a callus shaver. This video is available in the “Related Videos” section of the Full-Text article at PRSGlobalOpen.com or at http://links.lww.com/PRSGO/B3). After the cleaning, a swab culture of the periwound area found no microorganism growth.

A callus shaver is a simple option for the removal of persistent staining from the skin.

**Video Graphic 1.**
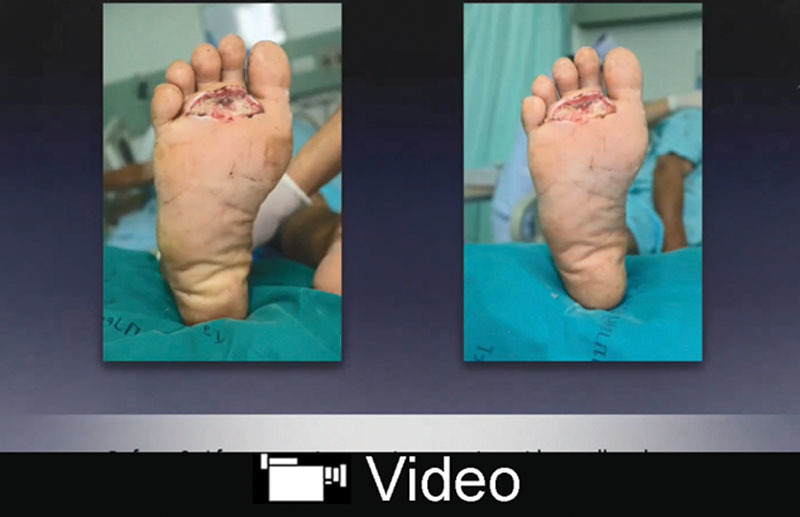
See video, Supplemental Digital Content 3, which demonstrates how to remove persistent staining with a callus shaver. This video is available in the “Related Videos” section of the Full-Text article at PRSGlobalOpen.com or at http://links.lww.com/PRSGO/B3.

## Supplementary Material
