# Supplementary figures and images for: Remove Persistent Staining with a Callus Shaver
Source: Plast Reconstr Surg Glob Open. 2019 Mar 25;7(3):e2140. doi: 10.1097/GOX.0000000000002140 (PMC6467618; doi:10.1097/GOX.0000000000002140)

โรงพยาบาลศรีนครินทร์ 25

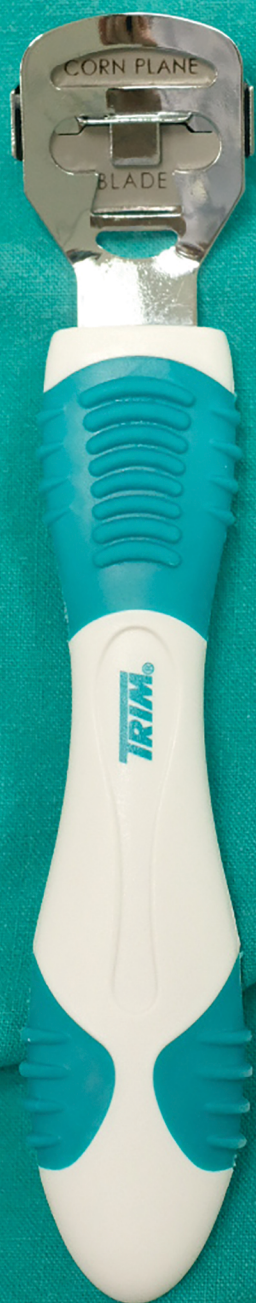

โรงพยาบาลศรีนครินทร์ 25

Supplement: Supplementary file 1 [file gox-7-e2140-s001.pdf]

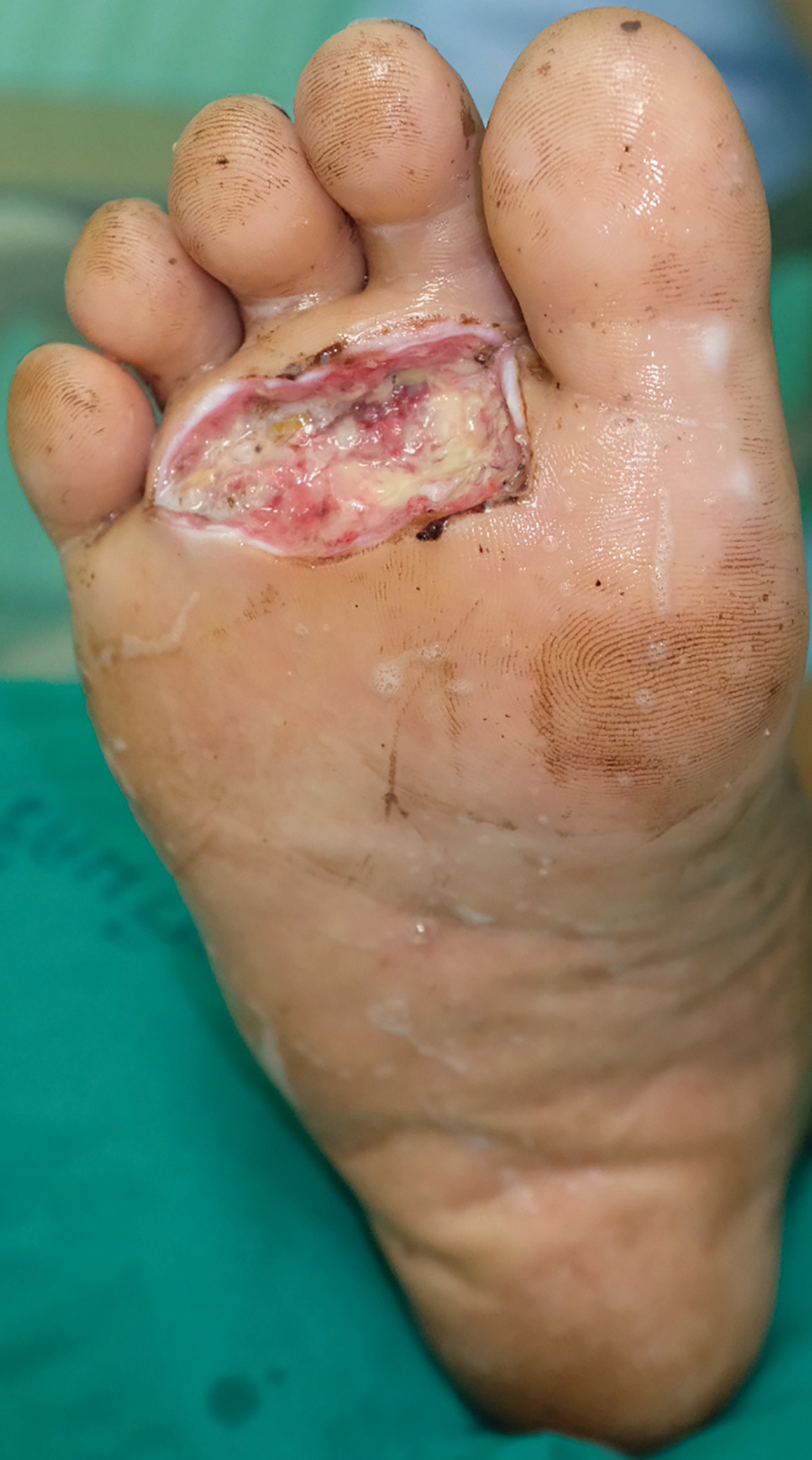

Supplement: Supplementary file 2 [file gox-7-e2140-s002.pdf]
